# The Port-Folio; or, Medical and Surgical Miscellany

**Published:** 1818-07-01

**Authors:** 


### FIFTH DEPARTMENT.

THE

### Port-Folio;

OR,

### MEDICAL AND SURGICAL MISCELLANY.

### I.

### *On the relative Importance of Morbid Anatomy, in reference to Therapeutical, Nosological, and Etiological Science.* BAYLE.

———— sunt certi denique fines,
Quos ultra, citraque, nequeat consistere rectum.

By some enthusiasts, morbid anatomy has been held up as the principal, or almost sole basis of nosology and therapeutics; while by others, who were not in the habit of cultivating this branch of medical science, the light of *post mortem* researches has been too much despised. It may not therefore be useles to enquire how far we are to depend on this guide in the healing art, and what are the just limits by which it ought to be circumscribed.

Diseases may be divided into two great classes, *functional* and *structural;* or vital and organic lesions. Most of the *symptoms,* or external manifestations of disease, belong to the *functional* class. Thus, suppose there be derangement, even of an *organic* nature in the lungs, it is by lesion of the *respiratory function* that it becomes cognizable to the senses. Suppose there be disease, organic or functional, in the heart; it is by disordered *circulation* that it is known. Those symptoms which are indicative of disordered *function* or vital properties only, are, for the most part, sudden in their accession, transient in their duration, and variable in their aspect; they leave no trace of their existence after death. Those, on the other hand, which manifest organic derangement are more *permanent*, and the *post mortem* appearances account for their order and degree of violence. We may designate them *physical* symptoms, remembering, however, that it is to the vital properties or *functions* they owe their origin. We frequently see functional, or vital, and physical symptoms combined in the same disease. Thus, in aneurism of the heart, the irregularity of the pulse, the palpitations, the sense of suffocation, the cough, &c. are all functional or *vital* symptoms. The actual increase of volume in the heart, which is perceived by the sight or touch, the dropsical effusions, the livor of the lips and countenance, &c. are *physical* symptoms. The derangements in the circulation, and in the respiration are *vital lesions;* the alterations of *structure*, &c. are physical *lesions.* These preliminary distinctions are necessary for the clear discussion of this important subject.

1.—Vital or *functional* lesions may be *primitive* and spontaneous. They by no means invariably determine an *organic* degeneration, even when they occasion death. But organic lesions are always *consecutive,* and cannot prove fatal but through the medium of *functional* derangement: in other words, there is no such thing as *primary* organic diseases; they are all subsequent to, and *dependent on,* previous lesion of the vital properties; *i. e.* disordered function, unless when resulting from an *external* cause.

2.—A distinction ought to be made between organic *lesions* and organic *diseases.* Under the *former* we comprehend all those morbid alterations in volume, texture, colour, &c. of parts, whether resulting from disease, or external cause, and of which the traces are manifest after death. We apply the term *organic disease* to those chronic affections which are essentially dependent on a serious and obstinate alteration of structure in any of the solids of the animal economy. Organic *diseases,* then, are the effect of organic *lesions;* and organic lesions are the result of *pre-existing* derangement of function, except where produced by external impressions. But be it remembered, that these organic diseases and organic lesions can only determine the sufferings, or produce the death of the patient, by re-acting, in turn, on the vital properties, and interrupting or destroying the due exercise of the *functions.*

3.—The above considerations will help to determine the utility of morbid anatomy, and fix the limits beyond which we have nothing to expect from it, in the science of disease. Dissection reveals not vital lesions, or disorder of *function*: it can only display to us the *effects* of diseases, and their *re-active* causes; but leaves us totally in the dark respecting those vital or *functional* lesions which constitute their essence.

4.—Morbid anatomy teaches us the physical derangements only. If we anatomically examine an aneurism, for instance, we find a sac filled with layers of coagulated blood, and a communicating aperture in the coats of the artery. But the original disease was not the aperture in the artery. It was a lesion in the *vital power or properties* of the artery, of which the aperture was an *effect* or consequence. The organic or structural lesion of the lungs, which we observe in those who have fallen under constitutional phthisis, is not the original disease: it was first produced by *vital* lesion; but arrived at a certain stage of developement, this *organic* lesion determines, in its turn, a multitude of vital or *functional* derangements, as difficulty of breathing, cough, hectic fever, emaciation, &c. and finally destroys the exercise of the functions entirely, and death is the result.

It is often impossible to ascertain the *primary* cause of these *vital* lesions that ultimately induce organic changes, excepting in those diseases derived from a specific contagion, as small-pox, measles, plague, &c. Here it is quite evident that the morbific cause *does* act on the vital powers and the functions, (although we are totally ignorant *how* it acts) and frequently superinduces *organic* lesion in its course. We may therefore form three series of diseases in the following order:

1.—Those diseases, the primary causes of which we know not, and which induce not organic lesions, but which present a similarity of symptoms that leads us to class them as different individuals of the same family, can be no other way distinguished than by the *physiological symptoms* which accompany them.

2.—When diseases are *contagious*, we advance a step in etiology, and form a class on the principle of *origin*, rather than symptomatology or *seat* of disorder. Thus the most *distinct* and the most *confluent* small-pox are ranged as only varieties of the same disease, however different their symptoms or the organic derangements that supervene.

3.—There is a class of diseases which are accompanied by a manifest lesion of the vital powers and functions, at the same time that they determine *organic* leisons; and it is in this series that morbid anatomy is extremely useful in ascertaining the nature of the accompanying structural derangement. On the two first series, morbid anatomy sheds but a feeble, if any light. But again, it is often extremely difficult to assign diseases their proper class; and here let us inquire how far morbid anatomy comes to our assistance in the classification.

We observe in different individuals, diseases which present the *same symptoms,* although they are by no means accompanied by the *same* organic lesions. Thus, two patients sink after a long chronic diarrhœa; and in one we find ulceration of the intestines, while in the other no organic change can be discovered. Of those patients who are long troubled with vomiting of brownish coloured matters, and ultimately die in a state of marasmus, a great proportion will, indeed, be found with scirrhus or ulceration of the stomach; but a certain number will exhibit no organic lesions in the viscus in question.

On the other hand, many patients die from the effects of a precisely similar organic lesion, whose symptoms, during life, were entirely different. Thus, of those patients in whom we find, *post mortem,* ulceration, or tubercles of the lungs, the greater number, it is true, will have exhibited thoracic pains, fatiguing cough, purulent expectoration, hectic fever, night sweats, &c. in their progress to the grave; but a certain proportion will show no such symptoms; or cease to experience them long before death. These are the difficulties which embarrass the *clinical* physician in his nosological arrangements, though they are easily put aside in the closet or in books. Thus if we class by *symptomatology,* we must place in the same species, chronic spasmodic vomiting, and scirrhus of the stomach; chronic catarrh, and tubercular consumption, &c. While, if we arrange on the foundation of *morbid anatomy,* we must, in a great proportion of cases, defer the classification till after the death or the recovery of the patient! Thus when two patients have, for several months, experienced difficulty of breathing, palpitation of the heart, and irregularity of the pulse, succeeded by dropsical effusions, ending in death, with puffiness of the face, livor of the lips, &c. we shall confidently state that they both died of the same disease. But on examination after death, if we find in one an aneurism of the heart, with ossification or other disease of the valves; while in the other no organic disease of any viscus is to be seen, we shall soon acknowledge the error of our diagnosis. This is an occurrence which every observant physician must have repeatedly met with.

Nevertheless the diagnosis founded on pathological anatomy is, upon the whole, the surest; but ought not to be entirely relied on; for, unfortunately, this branch of medical science is yet in its infancy, and infinite mischief is every day done by hastily setting down as *causes* what were only the *effects* of diseases. Nothing but minute attention to, and minute histories of, the *symptomatology*, as well as the *post mortem* appearances of *individual cases*, can furnish materials for a sound pathological classification of diseases.

---

### II.

### *Gun-Shot Wounds of the Intestines.* By M. Biot.

1.—A soldier of the 44th Demi-Brigade, received, near Altof, in Switzerland, a gun-shot wound, wherein the ball entered the abdomen, in the linea alba, two inches below the navel. He was conducted to Lucern, where I had charge of the hospital. On minute examination, I found the ball under the skin near the transverse process of the first lumbar vertebra of the left side. It was cut down upon, and extracted, when a quantity of extravasated blood and fæcal matter issued from the wound. Fæcal matter also was discharged by the anterior wound. On throwing up injections *per anum,* the fluids of which they were composed escaped, sometimes by one, sometimes by both orifices at the same moment. By the 24th day, stercoraceous matter and glysters ceased to issue from the anterior wound, which quickly healed: and in six weeks the posterior orifice also closed.

Venesection, repeated *pro re nata*—cataplasms, fomentations, lavements, and the most rigid abstinence, were the only means employed.

2.—Sarbeuf, a soldier in the 10th Demi-Brigade of Light Infantry, was wounded, near Stutgard, in the *left* hip, by a musket bullet. The ball perforated the sacrum, traversed the rectum, and came out at the anterior and superior part of the *right* thigh. I saw the patient on the second day, when foul matters were passing from both wounds. They kept this double route for eight or ten days, during which time no stools were passed per anum. By perseverance in throwing up glysters, the natural passage was re-established; the wounds diminished gradually in size, and ultimately healed in less than three months. Several small portions of sacrum were discharged from the wound in the left hip, and from the anus, in the course of the cure.

3. *Peritoneal Abscess communicating with the Intestine and the external Surface of the Abdomen; with discharge of a Cylinder of Intestine.* Biot.——J. B. Matheen, 21 years of age, was received, in an emaciated state, into the Great Hospital of Besançon, in the month of March 1793, for the cure of a suppurating tumour between the anterior superior spine of the ilium and the umbilicus. In the vicinity of the tumour we remarked three cicatrices, which the patient informed us were the remains of as many abscesses which occurred there during the three preceding years, and which had discharged good pus, and healed without difficulty. The present tumour was poulticed; and when fluctuation was perceptible, it was opened by Mr. Morel, Chief Surgeon of the Hospital. The opening gave issue to a considerable quantity of well-formed purulent matter. The finger introduced into the abscess felt the peritoneum beneath, into which there was an aperture that admitted the point of the finger. The opening was enlarged with a bistoury; and by pressure, an increased discharge of pus was effected. The patient was kept as much as possible on his face, in order to facilitate the exit of the matter. During some days there appeared nothing remarkable. But the patient evincing some symptoms of worms, a vermifuge was administered by Mr. Morel; which, to our astonishment, brought away *two large lumbrici from the wound,* and several *per anum.* And now, for the first time, we found the dressings soiled with fæces, and the sore exhaling a fœtid smell.

The patient was soon reduced to a state of the greatest debility, and was obliged to be fed from hour to hour with light nutritious aliment. The natural passage was also kept as pervious as possible by means of glysters. In this state a new abscess formed, three or four inches from the former, and within an inch of the symphisis pubis. When opened, a moderate discharge of pus ensued. But six days afterwards, a membranous substance presented itself at this last opening, which was extracted; and on examination, proved an entire cylinder of the intestinal canal about seventeen lines, or better than two inches in length! An artificial anus, at the best, was expected; but it was rather feared, that death would ensue from the escape of fæcal matters into the general cavity of the abdomen. For some days after the discharge of intestine, the whole of the fæces pass ... by the inferior wound, and also some worms. Nevertheless, the natural passage became again pervious, and the stools began to pass per anum. The discharge by the wounds decreased gradually, the wounds themselves contracted, the strength of the patient recruited, and at the end of fifty-seven days he left the hospital cured.

---

### III.

### *Observations on Belladonna.* By M. Marc.

As this medicine is now coming into pretty general notice, it may not be unacceptable to the British Medical World to know what has been said of it several years ago on the Continent.

"Numerous experiments made during the last thirty years, on Belladonna, enable us to appreciate pretty correctly the advantages of this vegetable. Notwithstanding the assertions of M. Muench, and the observation of M. Bucholtz, of Weimar, who assures us that he cured a case of *hydrophobia* that had already unequivocally declared itself, by Belladonna, we are constrained to reject all prospect of success from this remedy in rabies canina. Its inefficacy in *cancerous affections* has been demonstrated by many testimonies, but particularly by the experiments of M. Rahn, of Zurich. It appears to have been successfully employed as an *anti-syphilitic*, in chronic cases, unattended by inflammation. M. Boettcher, of Konigsberg, exhibited a composition of belladonna and calomel in *phagedenic ulcers* of the throat and of the genital organs, with marked and rapid benefit. But it is in the class *neuroses*, that belladonna has evinced its greatest power, especially in epilepsy and mania. J. E. Greding, Physician to the Pauper Establishment at Waldheim, has been long employed in ascertaining the effects of belladonna in these diseases; and his experiments, published by his son in 1790, are interesting. Greding exhibited the powder of the plant, in cases of epilepsy, in doses from half a grain to a grain and a half, three or four times a day. Also a combination of powder and extract (three parts of extract to one of powder) in doses from three to ten grains in the twenty-four hours. But notwithstanding the apparent safety with which M. Greding administered the remedy, in these cases, we think the dose too large, since it occasioned in a young man a loss of vision for the space of twenty-one days. Administered to twenty-three maniacs, the belladonna did not effect a perfect cure in any one; but the force of the paroxysms was greatly moderated, and the morbid symptoms unequivocally ameliorated.

"I have myself (says Marc) employed the extract of belladonna externally, and with some success in *tic douloureux.* A physician of my acquaintance assures me that he has cured an obstinate and chronic neuralgic affection of the face, by the *internal* administration of this vegetable." "There are few medicines whose utility in hooping cough has been more decidedly proved, than Belladonna, and yet it is surprising, that medical men should be so negligent in making use of it. M. Schaeffer administered this remedy to infants of one, two, and three years of age, every two hours, with great advantage. Several physicians, among whom we may cite M. Hufeland himself, have, since that period, tried M. Schaeffer's method, and all unanimously agree, that belladonna may be considered as almost a specific in hooping cough. This also accords with my own experience in three most obstinate cases of the disease. At this moment I am giving the medicine to two young children afflicted with hooping cough, and with decided advantage. During the epidemic prevalence of this distressing disease at Augsburg, in 1810, M. Wetzler treated thirty children by belladonna, and cured them all in a space of time varying from eight to fifteen days from the commencement of the remedy. M. Wetzler gave the root of belladonna in powder, mixed with a little sugar, in doses of a quarter of a grain night and morning, to children under twelve months, and in relative proportions as the ages increased.

"The simplicity of this mode of treatment, its facility of application, even among the most indigent classes of society, and the little repugnance which children manifest towards the remedy, are all advantages which entitle belladonna to a more extended trial than it has yet obtained.—*Dict. des Sciences Med.* tom. 5.

### IV.

### ILEUS.

Ann Smith, aged 50, (March 22, 1818) had been ill four days with colic pains, attended by obstinate costiveness and vomiting. I found her in bed at 9 A.M. with a pot of stercoraceous matter ejected from the stomach; abdominal muscles irregularly but strongly contracted; countenance anxious; tongue dry; pulse about 90, rather full, but equable. She had been bled, and had taken various cathartics, vomiting following the exhibition of every medicine; the costiveness remained unmoved; the twisting pain near the umbilicus sharp at intervals. I ordered her five grains of solid opium, and a large blister over the abdomen, and a glyster composed of two ounces of common salt dissolved in two pints of cold water. In the evening, I found her free from pain, but no fæcal motion followed the return of the glyster; she had not vomited since she took the opium. I now ordered her ten grains of calomel and small doses of spirits of turpentine every hour, according to the following formula:

℞. Spiritus terebinthinæ dr. iij mellis despumati, mucilaginis acaciæ tenuioris aa dr. iv. cochleare minimum omni hora sumatur.

On the next day, I found that her bowels had been freely evacuated, the pain had not returned, and she took light nourishment without nausea or inconvenience. The recovery was complete.

W. P.

*Bristol, March* 28, 1818.

---

### V.

### *Practical Observations on Catarrhal Disorders, or Inflammation of the Mucous Membranes.*

( Chiefly from the French of RENAULDIN. )

There is no class of disorders to which the British Practitioner's attention should be more particularly directed, than the one under consideration—First, because it is the most *common;* secondly, because it is connected with the most *fatal;* and thirdly, because it is the most *neglected* complaint in the whole catalogue of human infirmities. The French have certainly got far before us in the *pathological anatomy* of the mucous tissues, and it behoves us to take a lesson from them occasionally on this important point.

The term catarrh has been applied to every inflammation, acute or chronic, of the mucous membranes; an inflammation which is always accompanied by an increased secretion on those surfaces.

At a first glance, the mucous tissues appear to be very numerous, as they line the greater number of the hollow viscera. But this multiplicity decreases, when we consider that they are continuations of one another, and only differ as respects their situation, or the organs over which they are spread. They may be divided into two great classes: the one extending from the mouth, the nose, and the eyes, through the aerial and alimentary passages to the anus, denominated the *pneumogastric* membrane: the other lines the interior of the urinary and genital organs of both sexes, and receives the appellation of *genito-urinary* membrane. These two classes have no immediate communication; and it rarely happens that any irritation spreads from the one to the other.

All mucous membranes present two surfaces; one adhering to their respective organs; the other free, villous, constantly lubricated by a peculiar secretion, and destined to be in constant contact with foreign bodies; as well those from without, the air, the food, &c. as those from within, for instance, the various secretions, &c. In short, these membranes seem designed by Nature to guard the viscera from the injurious impressions of those heterogeneous substances which traverse their interior, in the same manner as the skin preserves the external surface from the too violent action of those bodies which surround us. Moreover, the organization of these mucous membranes is the same as that of the skin, and they form, in reality, but one continuous expansion. An epidermis, much finer it is true than that of the cutaneous surface, a papillary body, a chorion, mucous glands, and numerous vessels, compose the structure of the mucous tissues. The nervous papillæ lie immediately under the epidermis, and are the seat of sensibility in the organs; while the glands are studded in the chorion or cellular structure, and continually pour forth a mucilaginous secretion, through imperceptible orifices, on the villous surface. It is to irritation in the excretory ducts of these little glands, that we are to attribute the more abundant efflux and secretion of fluids; and it is inflammation of the same which characterizes the class of diseases denominated *catarrhal*, and embracing the whole of the phlegmasiæ of the mucous membranes.

This identity of organization in the various mucous tissues, which modern anatomy has developed, exactly accords with the identity of diseases, of symptoms, and of terminations, in the same structures. Why then divide these catarrhal affections into as many species as there are organs covered or lined by the said membranes? The ancients, who were comparatively unacquainted with morbid anatomy, reduced them all to three, as may be learnt from the Salernian verses—

> Si fluat ad pectus, dicatur rheuma catarrhus;
> Ad fauces branchus, ad nares esto coryza.

*Etiology.* The immediate cause of all catarrhal affections is a keen excitation or inflammation of the mucous membrane of the part, determined, in general, by sudden atmospherical transitions of all kinds, with their consequences, suppressed or checked perspiration or transpiration. Sometimes these phlegmasiæ owe their origin to the retrocession of an habitual discharge, an eruption, the sudden healing of an old ulcer, the retrocession of itch, rheumatism, gout, &c.

*Symptomatology.* This is so well known as scarcely requiring to be noticed here. Pain, heat, tumefaction, and redness characterize these as well as other inflammations. But it may be observed, that the natural secretions of the mucous membranes, when in a state of inflammation, are first diminished or suppressed; followed by a more copious secretion, at the beginning colourless, ropy, acrid; afterwards, thick, opaque, bland, yellow, and even *puriform*.

*Terminations.* Catarrhal inflammations terminate in health, in other diseases, and in death. In the *second* instance, the chronic inflammation degenerates into one of a more grave, alarming, and dangerous tendency. The mucous tissues become disorganized in such a manner, that, instead of a mucilaginous fluid, they secrete a real *purulent matter*, and phthisis supervenes. In other situations, as the stomach, &c. the said membranes become thickened, indurated and even scirrhous or cancerous, with accompanying hectic fever and death. At other times, catarrhal inflammation terminates in a few days fatally, by suffocation, when seated in the aërial passages.

*Pathology.* On opening the bodies of those who have died of catarrhal inflammation, the internal surfaces of the mucous membranes are generally covered with a whitish, yellowish, or purulent secretion; on removing which the villous tissue appears inflamed, and the glandular follicles more or less engorged and swelled.

*General Treatment.* Seeing that catarrh is an actual inflammation of an important class of membranes, which though capable, under favourable, or indeed ordinary circumstances, of spontaneous cure, yet too often degenerate into inflammation and disease of the neighbouring viscera, it is incumbent on the practitioner to check the disease by moderate but steady antiphlogistic measures. The plan of trusting the cure of catarrhal affections to warm clothing, diluents, bathing the feet at bed-time, and some gentle antimonial medicine, in a country like this, where the lungs are so much predisposed to obstruction, is the cause of many thousand deaths by pulmonary phthisis! A moderate bleeding at the very beginning, saline purgatives, rigid abstinence, and antimonials, to arrest the velocity of the circulation, with tepid diluent drinks, should supersede the farrago of anti-catarrhal juleps, pectoral lozenges, and stimulating sudorific drinks at bed-time to force out a perspiration, which are too often thoughtlessly prescribed even by intelligent practitioners. In the phthisically disposed, leeches to the chest, blisters, digitalis, and the semicupium, would, if promptly applied, save many a valuable life from the ravages of subsequent phthisis. The removal of the disease, *in its infancy*, by these means, will be found infinitely preferable to the slow, and not always safe process of *expectoration.*

---

Sir William Adams having had the honour to be nominated by His Majesty's Government, to superintend that part of York Hospital, Chelsea, which has been appropriated to the reception of the blind Pensioners belonging to the Army, Navy, and Artillery, feels it a duty to lay open to the Profession at large his new modes of treating them. This duty is suggested as well by the peculiar confidence which has been reposed in him, as by the high sanction thus conferred upon his improvements in Ophthalmic Surgery. He therefore freely invites all Medical Practitioners and Students, who are interested in the advancement of this branch of Surgery, to attend his operations at York Hospital; which, for their convenience, will be performed in future on Tuesdays and Fridays, between the hours of seven and nine in the morning.

To remove all doubt or misconception, with regard to Sir William Adams's practice, he proposes, on each of these days, to give a description of the nature of one of the diseases to be operated upon; the general modes of performing the operation; his peculiar mode; and his reasons for deviating from the usual practice, where such deviation has been found necessary.

The records kept of each case, from the patient's admission into the hospital to his final discharge, will be open at the periods already mentioned, for the inspection of such gentlemen as attend; so that the Profession will be enabled fairly to appreciate the character of the *new* as compared with the *old* modes of practice.

It is expected that from *Fifteen Hundred to Two Thousand* Patients will successively be placed under the care of Sir William Adams in this Institution.

*26, Albemarle Street, June* 10, 1818.

*** A fresh set of blind Pensioners have been admitted into York Hospital within these few days.

OBITUARY. Died at Leeds, on Monday the 18th of May, of a typhus fever, one of the brightest ornaments of society, and most learned of the Profession, JOHN THOMSON, M. D. æt. 35, late of Halifax. If the virtues and acquirements which this worthy man possessed could so be pourtrayed as to excite the emulation of the present and rising generations, a more valuable legacy could not be handed to them. Possessing a superior mind, which precluded an ungentlemanly thought or deed, deeply versed in the classics, and straining every nerve to advance the honour and integrity of his profession, he fell an early victim to his professional duties, deeply lamented by all who had the pleasure and honour of his acquaintance.

At Edinburgh, on the 14th of June, in the prime of life, and the noon-day of his talents and utility, JOHN GORDON, M. D. F. R. S. E. Lecturer on Anatomy, and on the Institutions of Medicine, in Edinburgh. This lamented gentleman was cut off by typhus fever. However much we may have differed from him occasionally on scientific questions, we can honestly say that we deeply deplore his untimely fate, and that we are not slow to feel virtuous sorrow for talents that promised well to medical science, thus prematurely quenched in darkness.

In Hatton Garden, on the 20th of June, JOSEPH ADAMS, M.D. Physician to the Small-Pox and Vaccination Inoculation Hospital, Pancras, Lecturer on the Theory and Practice of Medicine, &c. A few days previous to his death, having quitted his carriage for the purpose of walking across the fields to his new residence near Holloway, he unfortunately stepped into a cavity of the ground, fell backward, and fractured his right leg; but was going on so well in the progress of cure, that he was pronounced to be out of danger on the day before his death. About two hours preceding his demise, he took his dinner with good appetite, and was remarkably cheerful, when a sudden and most unexpected change took place, of which he was so perfectly sensible that he dictated a prescription for himself, but ere the messenger returned, he expired. Dr. A. was the predecessor as well as the successor to Dr. Fothergill in editing the London Medical and Physical Journal, and well known as the Author of several works which have contributed to promote the knowledge of disease no less than to advance his own reputation. He was the son of a respectable Apothecary in Bishopsgate Street or Spital Square, and commenced, we believe, his professional life as a general practitioner; after which he resided several years as a physician at Madeira, where he was succeeded by Dr. Andrews. Shortly after quitting that island for the metropolis, he became, by the especial privilege granted to the President of the College of Physicians, a Licentiate of that body, the usual requisite of residence in a University having been dispensed with upon that occasion. As a Practitioner, Dr. A. maintained a most respectable rank in public estimation; and in his intercourse with his professional brethren, his conduct was always marked with the urbanity of a gentleman and the liberality of a scholar. He was a zealous defender of the doctrines of his master, John Hunter, and gave to the world Memoirs of the Life of that celebrated Anatomist and Physiologist; a second edition of which work, with additions, was in the press, and nearly finished, at the time of his decease. Dr. Adams's Treatise on Morbid Poisons, as well as his other productions, met with a very favourable reception; and, in addition to the several works he had already written, he was preparing a complete edition of all Mr. Hunter's publications, when the indiscriminate hand of Death arrested his career, and terminated all his earthly pursuits.

